# Perceptions of HPV vaccination and pharmacist-physician collaboration models to improve HPV vaccination rates

**DOI:** 10.1016/j.rcsop.2021.100014

**Published:** 2021-04-22

**Authors:** Benjamin S. Teeter, Catherine R. Jensen, Jeremy L. Thomas, Bradley C. Martin, Pearl A. McElfish, Cynthia L. Mosley, Geoffrey M. Curran

**Affiliations:** a Center for Implementation Research, Department of Pharmacy Practice, College of Pharmacy, University of Arkansas for Medical Sciences, 4301 W. Markham St., Slot 522, Little Rock, AR, USA; b College of Pharmacy, University of Arkansas for Medical Sciences, 4301 W. Markham St, Little Rock, AR, USA; c Division of Pharmaceutical Evaluation and Policy, Department of Pharmacy Practice, College of Pharmacy, University of Arkansas for Medical Sciences, 4301 W. Markham St, Little Rock, AR, USA; d University of Arkansas for Medical Sciences, 1125 N. College Avenue, Fayetteville, AR, USA; e Central Arkansas Veterans Healthcare System, 2200 Fort Roots Drive, North Little Rock, AR, USA

**Keywords:** Community pharmacy, Human papillomavirus, Vaccines for children, Pharmacist-physician collaboration, Adolescent vaccination

## Abstract

**Background:**

Human Papillomavirus (HPV) is the most common sexually transmitted disease in the United States (US), with 12 cancer causing strains. Vaccination rates in the southern US fall below the national average. Pharmacists provide an opportunity to improve vaccination rates.

**Objectives:**

The objectives of this study were to 1) identify barriers and facilitators to providing the HPV vaccine and Vaccines for Children (VFC) program participation in pharmacies and clinics, and 2) assess pharmacy staff, clinic staff, and parent perceptions of 3 collaboration models to improve HPV vaccination.

**Methods:**

A developmental formative evaluation was conducted with pharmacy staff, primary care clinic staff, and parents of adolescent children. Interview guides were informed by the Consolidated Framework for Implementation Research (CFIR). Barriers and facilitators to HPV vaccination and VFC participation were explored. Additionally, acceptability of 3 collaboration models were explored: 1) a shared-responsibility model in which a physician provides the first dose of HPV vaccine with the second provided in the pharmacy, 2) a pharmacy-based model in which a clinic refers patients to the pharmacy to receive both doses, and 3) an insourced model in which pharmacists schedule days to provide the vaccine in the collaborating clinic.

**Results:**

Twenty-nine interviews were conducted between August 2019 and June 2020. Both pharmacy and clinic staff had positive views toward the HPV vaccine and vaccinations in general. Pharmacists and physicians reported parental awareness and education as a barrier to HPV vaccination. Counseling about HPV vaccine was reported as being more time-consuming because of the stigma associated with the vaccine. Parents were willing to have their children vaccinated for HPV in the pharmacy but desired their child’s physician be involved in the immunization process. The shared-responsibility model was the most favored of the 3 collaboration models.

**Conclusion:**

Perceptions of the HPV vaccine and vaccination in pharmacies were positive. Collaboration between clinics and pharmacies to improve HPV vaccination rates is viewed positively by pharmacy staff, clinic staff, and parents. This study will guide implementation of pharmacist-physician collaborative models to improve vaccination through pharmacy participation in the VFC program and HPV vaccination.

## 1. Introduction

Human Papillomavirus (HPV) is the most common sexually transmitted disease in the United States (US). An estimated 19,200 women and 11,600 men are diagnosed with HPV-related cancer each year in the US, with cervical cancer being the most common.^1^ Cervical cancer is the fourth most common form of cancer in women around the world, and a leading cause of cancer death in women globally.^2^ There are disparities for racial, socioeconomic, and geographic subgroups, with HPV-related cancer incidence and death rates higher among black, low-income, rural women, particularly in the southern US.^3^

Many HPV-associated cancers are preventable through HPV vaccination. In the US, Gardasil-9 is recommended for children beginning at age 11 but can be given as early as age 9 and is typically recommended to individuals as old as 26.^4^ The vaccine is administered as a 2 or 3 dose series depending on age of vaccination initiation and the child’s immune system.^4^ Despite improvements in recent years, the HPV vaccination series completion rate for adolescents still falls short of the Healthy People 2030 goal of 80% with only 48% of adolescents aged 13 to 15 years having completed the series in 2018.^5^ Of particular concern are southern, rural states where adolescent HPV vaccination completion rates trend below the national average and are lower than metropolitan areas of the US.^6^

### 1.1. Vaccines for children program

Vaccines for Children (VFC) is a program administered by the Centers for Disease Control and Prevention (CDC) that provides free vaccines, including HPV, to providers for administration to adolescents at no charge. All children under the age of 18 and enrolled in Medicaid or uninsured are eligible. For administering the vaccine, providers earn an administration fee from the Centers for Medicare and Medicaid Services (CMS). Despite the strong reach potential afforded by access to free vaccines, participation in the VFC program is low, especially in the southern US. Low participation in the program by physicians in some rural areas results in wait times as long as 12–18 weeks for vaccination appointments.^7^

### 1.2. Pharmacy participation in VFC program

Pharmacists are established immunization providers in the US since first earning the ability to immunize in 1996.^8^ Nearly all states (45/50) allow pharmacists to administer HPV vaccine under an immunization protocol. The most common requirement is a general immunization protocol between a physician and a pharmacist that grants the pharmacist authority to administer vaccines to any child. Community pharmacies are highly accessible when compared to clinical vaccination sites due to their extended business hours, no copays for visits, and no requirement to schedule an appointment.^9–11^ For individuals living in low income and/or rural areas with few provider options, these characteristics may make pharmacies especially attractive. Pharmacies have had success in increasing access to other vaccines with an estimated 6.2 million additional influenza immunizations and 3.5 million additional pneumococcal immunizations attributed to pharmacies annually.^12^

A large national survey of 1504 parents of adolescents found 81% endorsed pharmacist-provided HPV vaccination as long as the pharmacist had received proper training, reported the vaccine doses to the adolescent’s primary care physician (PCP), and referred the adolescent to the PCP for other health services.^13,14^ Nevertheless, HPV vaccination in the community pharmacy setting has been limited. Previous research conducted in a southern, mostly rural state in the US found only 15.9% of pharmacies offered HPV vaccine; only 4.4% had administered at least 1 dose in the past year.^15^ Additionally, participation in the VFC program is extremely low - there were only 68 pharmacies enrolled in the VFC program nationwide as of December 2020.

### 1.3. Collaboration models

A variety of potential collaboration models between physicians and pharmacists could be developed that may facilitate an increase in HPV vaccination. For example, in a “shared responsibility model” an agreement between physician/clinic and pharmacist/pharmacy can be established so that the first dose of HPV vaccine is given in the clinic while the second dose is provided in the pharmacy.^16^ This collaboration model may be beneficial for pharmacy/clinic partnerships in which both are enrolled in the VFC program. Another example is a “pharmacy-based model” in which a pharmacist and physician agree that all adolescent patients will receive a strong recommendation in the physician’s clinic and all doses of HPV vaccine will be administered in the pharmacy. This collaboration model may be beneficial for pharmacy/clinic pairs in which only the pharmacist is enrolled in the VFC program. A final example is an “insourced model” in which a pharmacist and physician agree on specific days/times that the pharmacist provide immunizations in the physician’s clinic. This may also be a collaboration model that is beneficial for pharmacy/clinic pairs in which the pharmacist is the only VFC enrolled provider. Regardless of the model chosen, communication between pharmacy and clinic will be crucial to ensure accurate vaccination records are kept for physician and pharmacist follow-up and completion of the multi-dose series.

### 1.4. Study objectives

The objectives of this study were to 1) identify barriers and facilitators to providing the HPV vaccine and VFC participation in community pharmacies and clinics, and 2) assess pharmacy staff, clinic staff, and parent perceptions of the 3 collaboration models to improve HPV vaccination series completion rates.

## 2. Methods

This study conducted a developmental formative evaluation^17^ using semi-structured interviews with pharmacy staff, primary care clinic staff, and parents of adolescent children living in the southern US. A regional supermarket pharmacy chain agreed to partner with the research team for this study. The leadership of this pharmacy chain had already made the decision to dedicate resources to support the adoption and initial implementation of the VFC program. This allowed for the unique opportunity to explore barriers and facilitators beyond the adoption decision. In order to ensure multiple points of view were taken into consideration when determining barriers and facilitators, pharmacy staff interviewed included pharmacy managers, staff pharmacists, and pharmacy technicians. It was important to collect data from primary care clinic staff that practice in the same geographical area as our partner pharmacies and have shared patients. Pharmacy staff nominated primary care clinics and/or physicians for recruitment based on their number of shared adolescent patients. Leadership at nominated clinics was contacted and permission to recruit clinic staff was obtained. Once permission was obtained, clinic leadership suggested an initial staff member to contact for an interview. Additional staff members were identified using snowball sampling and asking, “Who else at your clinic would you suggest I contact to speak about HPV vaccine and potential collaboration models?” at the end of each interview. To ensure multiple viewpoints within the clinic were considered, physicians, advanced practice registered nurses (APRNs), clinical pharmacists, and nurses responsible for administering vaccines were invited to participate in an interview. Parents of children age 11–17 were also nominated by our partner pharmacies for recruitment. These parents were individuals that the pharmacist felt would be willing to participate based on prior interactions. Vaccination status of potential participants was not used to determine eligibility but was asked during the interviews. Potential participants were contacted via telephone to invite them to participate in an interview using a recruitment script. The University of Arkansas for Medical Sciences Institutional Review Board approved this study and classified it as Exempt, category 2 based on Title 45 CFR 46.101. A waiver of documented informed consent was granted given that some subject interviews would occur via telephone and mailing an informed consent document with identifiable names of subjects could compromise confidentiality. However, prior to the start of each interview, a verbal informed consent process with IRB approved script was followed in which the participants were given detail about the risks associated with their participation. Verbal consent was obtained from all interview participants. All interviewees were offered a modest monetary incentive for their participation.

### 2.1. Data collection

Interview guides were informed by the Consolidated Framework for Implementation Research (CFIR).^18^ This framework borrows constructs from Rogers’ Diffusion of Innovations,^19^ Greenhalgh and colleagues’ review of diffusion of innovations in service organizations,^20^ and other sources, to organize a multitude of constructs into 5 domains: 1) intervention characteristics, 2) outer setting, 3) inner setting, 4) characteristics of individuals, and 5) process. Using CFIR as a guide, interview questions explored barriers and facilitators to HPV vaccination, other services provided in pharmacies and clinics, vaccination procedures, and frequency of vaccine administration. Example interview questions utilized to explore CFIR domains are provided in Table 1. Pharmacies and clinics were also asked about current participation in the VFC and their thoughts on the implementation of this program in their practice settings. Questions about the 3 collaboration models were included in each interview with pharmacy staff, clinic staff, and parents. Pharmacy and clinic staff were asked whether they would be interested in participating in a collaboration, which of these models they would prefer, and what barriers may hinder implementation of these models. Parents of adolescents age 11–17 were asked about their perceptions of HPV and other immunizations, their relationship with their physician and pharmacist, whether they would have their children vaccinated at their pharmacy, and which collaboration models they preferred. All interviews were conducted by a PhD-trained member of the research team with expertise in qualitative data collection using interviews. All interviews were digitally recorded for analysis.

### 2.2. Data analysis

Recorded interviews were analyzed using a rapid content analysis technique in which summary templates are utilized to code key excerpts to preidentified areas of interest.^21^ This method allows analysis to take place immediately following completion of interviews without the need for full transcription. Researchers listen to audio recordings of interviews and only transcribe statements from participants that fit within one of the summary template areas of interest. This allows theme saturation to be identified while data collection is ongoing and as a result, reduces unnecessary data collection and ensures timely dissemination of findings. Summary template areas of interest reflected the 5 CFIR domains listed in section 2.1. Interview statements of interest were transcribed and coded to a specific CFIR domain and classified as a barrier or facilitator. Additionally, summary templates included areas for interpretation of responses to questions about the 3 collaboration models. Two researchers performed the coding process. Summary templates with transcribed interview excerpts were uploaded to MAXQDA qualitative data analysis software^22^ for comparison. Discrepancies between coders were resolved through discussion.

## 3. Results

Interviews took place between August 2019 and June 2020. A total of 29 interviews were conducted. Fourteen pharmacy staff (8 pharmacists, 6 technicians) from 5 pharmacies, 10 clinic staff (5 physicians, 2 APRNs, 2 nurses, 1 clinical pharmacist) from 3 primary care clinics, and 5 parents of adolescent children participated in an interview. Individual recorded interviews lasted between 20 and 60 min.

### 3.1. Barriers and facilitators reported by pharmacy staff

Intervention characteristics were more commonly seen as facilitators than barriers in pharmacy interviews. Since vaccinations were already provided in the participating pharmacies, many expressed that there was little difference between what they are currently doing and what would be required if they were asked to increase the HPV vaccinations. Pharmacy staff explained, “We’re used to giving shots and do it pretty well. We give the flu shot, and Shingrix, pneumonia, Tdap… It’s not hard to step away and do that for a minute.” Another pharmacy staff member stated, “It would be roughly the same as we do for flu. We could advertise a little and see.” They also felt focusing on increasing HPV vaccination would not result in a large increase in their workload. One pharmacist explained, “Even when we advertise, outside of flu season, we only do a handful of injections a month.” When asked if they would need additional training or education to provide the HPV vaccine, the majority of pharmacy staff felt they were adequately trained but may benefit from education materials they could share with patients to start the conversation and provide information. When asked specifically about the VFC program, some pharmacists felt the requirements of the program were somewhat of a barrier. Many pharmacists mentioned the requirement of a second refrigerator for VFC vaccine storage and a secondary emergency location in the event of a refrigerator malfunction as barriers to participation but, “once you have that and you get the hang of the reporting, it takes about 30 minutes to an hour of extra work every month to make sure everything with the program is how they want it.”

Barriers in the outer setting were more frequently mentioned than facilitators in the pharmacy staff interviews. The majority of interviewed pharmacists viewed misperceptions of the HPV vaccine, along with other vaccines, as barriers to education and provision of the vaccine. One component of the misperception was associated with advertisements for the vaccine and sexual transmission. For example, one pharmacist stated, “it needs to be advertised as a cancer preventer and not a sexually transmitted disease.” Pharmacists indicated they believed the pharmacy was a suitable location to administer the HPV vaccine but noted there seemed to be a lack of demand or knowledge for the vaccine. For example, one pharmacist stated, “There are probably more [adolescent patients] than I realize but our population is older.” Another pharmacist echoed this sentiment saying, “… we have an older population and not in a prime location for children. We’re mostly retirement age.” When asked specifically about the VFC program, the majority of pharmacy staff reported that there were a large number of rural, low-income families in their area, and the children in those families would likely be eligible but that the “medical clinics usually see the younger patients.” Overall, pharmacists expressed a need to increase awareness, educate parents on the vaccine, and make sure families know they can have their children immunized quickly and for free in the pharmacy.

Pharmacies reported multiple barriers and facilitators in the inner setting. Of note, structural characteristics, such as lack of a private room for vaccine administration, were reported. One pharmacist explained, “There could be a privacy thing. It might make people a little uncomfortable… especially children.” There was clear support from upper management through the decision to enroll in VFC but some of the pharmacy staff were not as enthusiastic. For example, one pharmacist explained, “Our MTM [medication therapy management] program is pretty intensive and adding that on top of everything…I’ve heard some frustration. But I think we’re still positive…we as our company as a whole… that’s the direction they want to go and we’re following as best we can.” Pharmacy staff highlighted the support they receive from upper management with one pharmacist stating, “It’s a few hours of CE [continuing education] every year and they give us time to get that completed… the company covers stuff like that.” Additionally, the overwhelming majority felt their pharmacies were great places to make vaccination a priority. One pharmacist said, “[The pharmacy] is one place I think it should be administered, but it’s not happening yet…conversations mostly happen at physicians’ offices… but we should be having those conversations in the pharmacy, too.”

Overall, the characteristics of the individuals working in the pharmacies were considered by participants to be facilitators more frequently than barriers. Responses to questions found that the majority of pharmacy staff interviewed had positive attitudes toward vaccination and demonstrated broad support for vaccine administration in their pharmacies. Many pharmacy staff said they had their own children vaccinated for HPV and they would have no problem recommending the vaccine to parents of adolescent children. Pharmacists and pharmacy technicians reported being knowledgeable about the HPV vaccine and pharmacists were confident in their ability to provide the vaccine and counsel patients. One pharmacist stated, “I think pharmacists are perfectly capable of doing the kind of education related to disease prevention that physicians can do.” The majority of pharmacists did not view the HPV vaccine as different from other vaccines but mentioned public stigma associated with the HPV vaccine as a concern. For example, one pharmacy staff member stated, “It’s [the vaccine is] harder sell because it’s not as commonly discussed in regards to the disease it’s actually preventing.”

Process factors mentioned by pharmacy staff included use of opinion leaders, especially management within the company. Administration in the pharmacy was typically described as “top down” decision making with support provided by management to implement decisions. For example, one pharmacy staff member explained, “…[management] really support us doing these things… these new services. [Manager] helps us find help to get started and points us in the right direction with how to track and record…” Overall, pharmacy staff felt they were given the resources and support from management needed to successfully provide HPV vaccines and participate in the VFC program.

When pharmacy staff were asked about the potential collaboration models, all pharmacy staff interviewed were supportive of collaboration between the healthcare settings. The majority of pharmacy staff indicated positive opinions toward collaborating with a primary care clinic and were supportive of either the shared-responsibility or pharmacy-based model. There wasn’t much interest in the insourced model with one pharmacy staff member explaining, “…the logistics… just scheduling and transporting the vaccine and keeping it stored at the right temperature… I’m not sure if the VFC program would even let us.” The majority of pharmacy staff favored the shared responsibility model. One pharmacist said pharmacists could serve as an “option when patients can’t get into the doctor’s office and need vaccines for school.” Another pharmacist explained, “Our patients really trust us but they also really trust their physicians… I think it would be really beneficial if they had that recommendation from the physician to get it at the pharmacy… and they wouldn’t have to go to the health department.” Pharmacy staff also felt they could leverage their existing relationships with local clinics with one pharmacist stating, “I think it’s an excellent idea. I have no problem reaching out to those physicians. I know those clinics and talk to the nurses and doctors… And most of them, they’ve quit giving all those shots. They’re kind of referring them all out… that’s what I’ve seen.”

### 3.2. Barriers and facilitators reported by clinic staff

The majority of primary care clinic staff interviewed explained that providing immunizations was part of their daily routine. One clinic staff member explained, “We do pretty much the entire series of vaccines from birth to the adult recommended vaccines.” When asked specifically about the HPV vaccination, clinic staff explained that, “there is no accountability on the patients end to have that completed… We offer it but parents often say they are not interested. It is difficult to respond. Most of the time we hear that they just don’t need it. We give them the background of the importance of the HPV vaccine, the cancer prevention and all that, but they say they aren’t interested.” The majority explained a big difference between counseling on HPV vaccine and other vaccines is that some parents believe it will lead to sexual activity. One physician explained, “They think that by vaccinating their children against HPV that you’re somehow endorsing promiscuity and they obviously don’t want to do that.” Many physicians also felt that the effort to provide the vaccine was greater than other vaccines because it is a multi-dose series. One key informant explained, “Since it is a series of 2 or 3 shots, we may be successful in giving the first dose, we give the patient a reminder card with a date on it, but most of them don’t follow the recommendation.”

In the outer setting, a large number of barriers were reported by clinic staff. Education and trust were seen as major barriers to vaccines in general but especially when considering the HPV vaccine. One clinic staff member explained, “In just the past few weeks I’ve come across a few parents who chose not to vaccinate their children. I asked them why and the only reasons I could get from them were ‘personal beliefs’ which makes it hard to understand where the beliefs come from or how to address them… And I don’t want to push someone into something they don’t feel comfortable doing. I ask them to think about it or talk about it with their spouse and call me with any questions.” Another physician explained their clinic’s policy, “we have printed materials for distribution and all of it is at an 8^th^ grade reading level or below. We try to make it easy to understand. We know there is misinformation about vaccines and anti-vaxers and our population has low health literacy and I think that contributes to them being easily influenced or tricked into believing all the negative stuff on social media and Facebook.” Other parent resistance toward the HPV vaccine was explained by one clinic staff member who explained, “Honestly, [I have] more difficulty convincing parents with the HPV vaccine. There is a misunderstanding and it does take time to explain. It’s associated with something that can be sexually transmitted, there is sometimes a little bit of reluctance from their perspective because it maybe condoning or allowing a larger amount of sexual activity. Because of that, there’s a little bit of resistance we sometimes meet with that set of vaccinations. It’s a challenge we’ve seen for a time.” Additionally, the lack of a mandate for the HPV vaccine was viewed as a barrier to many physicians. One physician explained, “We feel it is an essential component of the immunization schedule but it is not made mandatory through the school systems like the other vaccines.”

For the inner setting, the majority of primary care clinic staff explained that HPV series completion rate was a “performance metric” (meaning something that they are measured on by their healthcare systems/insurers), although it was not one they thought they performed very highly on. While it was clear immunizations were a priority for the staff interviewed, they explained that leadership within their organizations often prioritize numerous activities at once and it can be difficult to keep up with them all. Overall, clinic staff expressed the opinion that the clinic was the obvious place to have children immunized. One physician explained, “Parents bring their kids here to get their children vaccinated so they can go to school. When they bring them here, they kind of expect it. I think a lot of parents think that is the only reason for the well-child appointment.” The majority of the clinic staff interviewed reported that their clinics were enrolled in the VFC program but explained that the enrollment and monitoring requirements were handled by administrators, not by providers. One physician explained, “There’s only a small number of us here but that’s not something I deal with. I’m not sure who does that but it’s probably [administrative position]. VFC or not, it makes no difference to me.”

Regarding the characteristics of the individuals, clinic staff had a positive attitude toward vaccines in general, the HPV vaccine, and the VFC program. A majority also expressed a strong belief that vaccines are vital for all children to avoid unnecessary disease. One physician explained, “When a parent has concerns, I take the time, an extra 5 to 8 minutes, and counsel them on the importance of the vaccines. My appointments are scheduled for 15 minutes but I’ll mess up my whole day trying to explain this. In the real-world, if every parent is apprehensive, it won’t work… and sometimes you don’t have that much time to sit and talk to them in detail, but I try.”

Clinic staff did not have strong opinions on process factors such as opinion leaders or champions. Many mentioned performance metrics as ways they receive feedback but how they used that information varied. A small number of clinic staff reported benchmarking for improvement but for most, the metrics appeared to be more of a measurement of their patients’ acceptance of their recommendations, not of the effectiveness of their recommendations. For example, one clinic staff member said, “I know we track that… I know we want the number high… I’m not sure if they’ve [administration] done anything to try to improve or not.”

When clinic staff were asked about the 3 potential collaboration models, overall sentiments were positive. All clinic staff interviewed expressed interested in collaborating with a pharmacy to provide more HPV vaccines but there were some concerns about documentation and accuracy of records. For example, one physician explained, “Many times my adult patients can get vaccines elsewhere and I don’t ever know that it happened. And we’re within driving distance of [bordering states] so we see patients from 3 different states. And when it happens state to state, that’s when you run into problems. So, when I start talking to them about it, they’ll say they’ve already had that… that is why documentation is extremely important. It needs to be in the vaccine registry or we need to be informed somehow. We used to get a ton of faxes but that has kind of stopped.” The shared-responsibility model was overwhelmingly the favored model among all interviewed physicians. Frequently mentioned reasons that physicians favored this model over the others were that vaccines provide an incentive for children to visit the clinic for well-child exams and that they felt it was important that they provide initial counseling about HPV and HPV vaccine. One physician stated, “If the vaccines were just administered at the pharmacy that would decrease the number of opportunities for the parents to bring their children in for wellness visits. That is what I think they think about when they bring their children in, the shots, they’re not thinking about the other advantages of the physical signs, searching for signs of depression, diabetes… I don’t really like the [pharmacy-based model] very much.” Another physician explained, “I think the most effective would be the shared-responsibility… I think it’s still important to have everyone involved with it. I think that one would be more effective, especially with the 2-part vaccination. It’s a nice option that patients would welcome. People don’t like going to the doctor so if you can avoid that and go to a pharmacy in the community which many times happens to be in a store they’re already going to, you’re more likely to capture that repeat.” There was also minor concern with how administration at some of the clinics would react. For example, one physician explained, “I think it’s a brilliant idea. As long as the systems are updated and there is a way for us to know what vaccines have been given…the issue would be more with administration than anything.” Additionally, there were some concerns that partnering with a specific pharmacy may upset other pharmacies in the surrounding area. One clinic staff member explained, “We have local community pharmacies that do great things for our patients. they’ve really stepped up and go way beyond what is expected. If they hear we are sending all these patient somewhere else to get this… I don’t know. I think that’s going to take some political savviness or good communication… That we aren’t trying to get them to transfer all their ‘scripts to another pharmacy and that we’re just trying to get them their HPV.”

### 3.3. Parent perceptions of the HPV vaccination and collaboration models

Overall, the majority of parents indicated a positive perception of vaccines. One parent said, “I am pro-vaccine…I think vaccines are important and I think all kids should get them.” Another parent explained, “If they’re recommended by a professional, then, I mean, I’m pretty sure the professional knows what they’re talking about.” All but 1 of the parents reported that their children had received the influenza vaccine the previous year. When asked specifically about the HPV vaccine, the majority of parents reported not having their children immunized. When asked why not, the majority of parents stated that it had not been recommended or that they believed their children were too young. One parent explained, “I know it’s a newer vaccine… It’s not one that I got… So I had sort of a negative stigma towards it but…as a parent, you can do as best as you can and try to encourage your kids to make the best decisions they can but you don’t always know what they might encounter if they only have 1 sexual partner or multiple sexual partners or things like that… so, like I said, I think my kids are too young right now but if they were not, we’ll do the vaccine.” Another parent explained, “they’re just, their younger and not sexually active so we’re waiting to get that one.” Another explained, “We haven’t talked about that one yet but when it’s recommended, we’ll talk about it and they’ll probably get it.” When asked whether they would be willing to have their children receive their immunizations in the pharmacy, parents responded favorably. One parent said, “…the pharmacist knows just as much about the vaccine as the doctor or they wouldn’t be able to do it, right? So, I wouldn’t have a problem with that. The only time it would be negative is if they had to get other vaccines… so you’d have to go to multiple appointments. That would be the only negative downfall.” This view of pharmacists as vaccination providers and the pharmacy as an appropriate vaccination location was expressed by another parent who had previously received an influenza vaccine in the pharmacy. They stated, “It was easy, fast, I didn’t need to have an appointment. If I could do it at the pharmacy without an appointment, I would do that every time. I’m not gonna lie, if it’s something like Tylenol [acetaminophen], I’ll buy it at Wal-Mart because it’s cheaper, but for everything else, I know my pharmacist and it’s convenient and I just, I like them.” The majority of parents still emphasized the importance of having their child’s physician involved in the process. For example, one parent stated, “I personally just found a pediatrician that I really respect, and so I look to her for guidance and I know she’s pro-vaccine and HPV vaccine…I’m going to let the doctor we’ve chosen, and trusted help, guide us in those decisions. If she says my children should get the vaccine and it doesn’t matter if they get it at her office or the pharmacy or wherever, I trust her.” When asked about the 3 potential collaboration models, parents expressed interest in the shared-responsibility or pharmacy-based model but explained the importance of including the physician. For example, a parent stated, “If I’ve already gone through and figured out the risk and feel like I don’t have any more questions and the doctor says it’s fine, then I think I’d feel comfortable going to a pharmacy.” Another parent expressed similar feelings saying, “I’m not opposed to that at all. As long as Dr. [redacted] knows about it, if I received a call from the pharmacy or from Dr. [redacted]’s office that said, hey, you know, your child needs this vaccine and you’re not going to get in here for a few weeks, but, you know, you can go ahead and get it at [pharmacy name], nuh-uh, I wouldn’t have any problem with that at all.”

## 4. Discussion

HPV vaccination completion rates fall short of national goals, especially in the southern US. Pharmacists are established immunization providers and have the ability to participate in the VFC program, but less than 100 pharmacies are enrolled nationwide. Previous research found the storage and handling requirements of VFC vaccines were a potential barrier in a chain pharmacy setting.^23^ Interestingly, this study also found storage and handling requirements were a barrier, but only during the initial implementation of the VFC program. Once enrolled, pharmacy staff reported that maintenance and documentation required only 30 to 60 minutes per month and VFC requirements were not disruptive to normal workflow. It is important to emphasize this finding in order to increase pharmacy enrollment in the VFC program.

To our knowledge, this was the first study to explore pharmacy staff, primary care staff, and parent perceptions of pharmacists as HPV vaccine providers. Interviews with parents suggest that they are willing to have their children receive the HPV vaccine in the community pharmacy, and they are confident in the training and abilities of their pharmacist. This supports previous research on parental acceptance of HPV vaccine provided by community pharmacists.^24^ Additionally, primary care clinic staff are supportive of pharmacist involvement in vaccinations. This finding supports previous research that found physicians were in favor of vaccination sites outside of a physician’s practice to improve influenza vaccination rates.^25^ This study suggests that parent and physician support for pharmacists as immunization providers for adolescent children reaches beyond influenza to all childhood vaccines as long as communication and accurate documentation procedures are in place. This finding reiterates the importance of accurate, up-to-date immunization registries and the need for development of a shared electronic medical record that provides access across practice sites and types.

Physicians indicated a desire to be involved in at least one component of the child’s HPV vaccination process; either through the initial clinic visit and recommendation of the HPV vaccine or by providing the first dose of the vaccine and vaccine education. Parents also stressed the importance of keeping their child’s physician involved. As a result, the shared-responsibility model was the most highly favored of the 3 proposed models among primacy care clinic staff and parents. It is clear from previous research that a strong recommendation from a physician is a predictor of parents willingness to have their children vaccinated.^26^ However, these recommendations do not appear to be occurring as frequently as needed; all parents interviewed had adolescent children eligible to receive the HPV vaccine but the majority reported it had never been recommended. Since the number of visits an average person makes to a pharmacy is significantly higher than trips to a physician’s clinic, pharmacists should take advantage of these interactions and recommend vaccinations for parent and child.

This study has several limitations. First, this study utilized a relatively small sample size of pharmacists, physicians, and parents from a single state in the southern US. Therefore, results may not be generalizable to other states or regions that have different immunization and scope of practice laws in place. Risk of selection bias should be considered when interpreting this study’s findings. The opinions of the those interviewed cannot be broadly generalized to a greater population, but instead serve as a guiding point in the formation of these collaborative models to improve future vaccination efforts. The interviewed parents were nominated by their pharmacist to participate in the study. This personal connection may have influenced their answers. Additionally, despite extensive effort, recruitment of parents proved to be difficult. Many parents who declined our invitation to participate said they felt overwhelmed due to the corona-virus pandemic requiring them to care for their children while working from home and that they did not have the time or energy to participate. We also did not collect participant demographics and it is likely that the parents who participated were not representative of the population of parents of children age 11 to 17. While the small sample of parents is a limitation of this study, themes from interviews were consistent with previous research.^24^ Finally, pharmacists, physicians, and parents were aware that they were speaking to a pharmacy researcher which may have introduced social desirability bias in their responses.

## 5. Conclusion

This study utilized an implementation science framework, CFIR, to guide examination of barriers and facilitators to HPV vaccination and community pharmacist as HPV vaccination providers. Collaboration between primary care clinics and pharmacies to improve HPV vaccination rates is viewed as a promising method by pharmacy staff, clinic staff, and parents. The shared-responsibility model was viewed most favorably due to the ability of this model to maintain involvement of a child’s physician while providing an alternative vaccination site that may offer improved convenience for the patient. This study will guide future research on the implementation of pharmacist-physician collaborative models to improve healthcare access through pharmacy participation in the VFC program and HPV vaccine administration.

## Supplementary Material

Interview Guides

## Figures and Tables

**Table 1:** CFIR domains and example interview questions.

Domains	Example interview questions
Intervention Characteristics	• What do you think about offering HPV vaccine in comparison to other vaccines? • What are your feelings toward the amount of time and energy that would be necessary to provide and be reimbursed for the HPV vaccine? • What are your perceptions of the cost required to provide the HPV vaccine?
Outer Setting	• What issues are you confronted with that are population/area specific? How do you overcome them? • What external factors/policies make offering the HPV vaccine more/less difficult than other vaccines? • What do you think the perception of your pharmacy/clinic is among the community? What about among parents of adolescent children?
Inner Setting	• What services are currently offered for the adolescent patient population? Are these different than the services offered for other patients? How? • What are your thoughts about the training and support you receive for the current services you offer? What about for new services? • Would pharmacy/clinic leadership be involved? How? • Are there structural characteristics of your organization that make providing vaccines easy/difficult?
Characteristics of Individuals	• What would you like to see included in the training to provide the HPV vaccine/VFC vaccines that would make it easier to implement the collaboration between your pharmacy and the physician’s office? • Do you feel like your pharmacy/clinic is an appropriate place to provide the HPV vaccine? Why or why not? • In general, what do you think about providing vaccines? What about the HPV vaccine? • Are your coworkers positive or negative toward vaccines in general? What about the HPV vaccine?
Process	• Do you believe you will encounter any pushback from any of your coworkers if you entered a collaboration with a pharmacy/clinic? How would you overcome it? • How do you know if you’re doing a good job when it comes to vaccinations? Other services? • What is the process like when making the decision whether to implement a new service? • What kinds of resources would you need to implement a collaboration with a pharmacy/clinic?
