# Supplementary material for: Perceptions of HPV vaccination and pharmacist-physician collaboration models to improve HPV vaccination rates
Source: Explor Res Clin Soc Pharm. 2021 Apr 22;2:100014. doi: 10.1016/j.rcsop.2021.100014 (PMC8856661; doi:10.1016/j.rcsop.2021.100014)
Supplement: Supplementary file 1 — Supplementary data: interview guides [file mmc1.pdf]

PHARMACY INTERVIEW GUIDE

HPV Pharmacy Site Interview Guide *(Based on Consolidated Framework for Implementation Research, CFIR)*

- Aside from dispensing medications, what services do you offer in your pharmacy for patients?
- What vaccines do you currently provide? How often?
  - How are they provided? In workflow? Specific pharmacist's role?
- In general, what do you think about delivering HPV vaccinations in your pharmacy?
- What do you think about the VFC program?
- What do you think would work most easily in this pharmacy to provide the vaccine? What wouldn't?

Intervention Characteristics

- What do you think about offering HPV vaccine in comparison to other vaccines?
- What are your feelings towards the amount of time and energy that would be necessary to provide and be reimbursed for the HPV vaccine?
- What are your perceptions of the cost required to provide the HPV vaccine?

Outer Setting

- What areas do you feel are most important to focus on in this community?
  - For adolescent patients? Underserved patients?
- What issues are you confronted with that are population/area specific?
  - How do you overcome them?
- What do you believe the perception of your pharmacy is among the community?
  - The Medicaid population in the community?

Inner Setting

- How do you believe the implementation of a collaboration between your pharmacy and a local physician or clinic would be received?
- What services are currently offered in your pharmacy for the adolescent patient population? Medicaid population?
  - Are these different than the services offered for other patients? How?
- What are your thoughts about the training and support you receive for the current services you offer?
  - What about for new services?
- Who do you believe would need to be involved in the process of offering the HPV vaccine through a collaboration with a physician from start to finish?
  - Roles and responsibilities?
- Would pharmacy leadership be involved? How?

Characteristics of Individuals

- What would you like to see included in the training to provide the HVP vaccine/VFC vaccines that would make it easier to implement the collaboration between your pharmacy and the physician's office?
- Do you feel like this pharmacy is an appropriate place to provide the HPV vaccine?
  - Why or why not?

Process

- Do you believe you will encounter any pushback from any of your coworkers when this is implemented?
  - How would you overcome it?
- How do you know if you are doing a good job?
- What tools would you need to implement HPV vaccines here?
- What is the process like when making the decision to offer a service like this?
  - Are individuals identified to lead the effort?
    - If so, how are they chosen?

Collaboration Model Questions

- What do you think about the collaboration models?
- Which model do you think would be most successful? Why?
- What barriers do you see to implementing one of these models?

General Closing Questions

- If you were offered the chance to implement the collaboration now that you've heard all this, would you be interested?
  - Why or why not?
- What would it take for you to be interested?
- What physician or clinic in the area would you want to collaborate with? Why?

CLINIC INTERVIEW GUIDE

HPV Clinic Interview Guide *(Based on Consolidated Framework for Implementation Research, CFIR)*

- What does your patient population look like?
- What vaccines do you currently provide? How often?
- In general, what do you think about the HPV vaccine?
- What do you think about pharmacists providing vaccinations?
- What do you think about the VFC program?
- What would allow you to be able to provide more vaccinations to adolescents?

Intervention Characteristics

- What do you think about offering HPV vaccine in comparison to other vaccines?
- What are your feelings towards the amount of time and energy that would be necessary to provide and be reimbursed for the HPV vaccine? VFC?
- What things would make offering the vaccine desirable? Is HPV vaccination rate a current performance metric for your clinic?

Outer Setting

- What areas do you feel are most important to focus on in this community?
    - For adolescent patients? Underserved patients?
- What issues are you confronted with that are population/area specific?
    - How do you overcome them?
- What do you believe the perception of your clinic is among the community?
    - The Medicaid population in the community?

Inner Setting

- How do you believe the implementation of a collaboration between your clinic and a local pharmacy would be receive by your staff? Your patients?
- Do you have a disease state management protocol with any pharmacies? If so, what does it cover?
- What services are currently offered in your clinic for the adolescent patient population? Medicaid population?
    - Are these different than the services offered for other patients? How?
- Who do you believe would need to be involved in the process of offering the HPV vaccine through a collaboration with a pharmacy from start to finish?
    - Roles and responsibilities?
- Would clinic leadership be involved? How?

Characteristics of Individuals

- What would you like to see included in the training to provide the HVP vaccine/VFC vaccines that would make it easier to implement the collaboration between your office and the pharmacy?
- Do you feel like this pharmacy is an appropriate place to provide the HPV vaccine?
    - Why or why not?

Process

- Do you believe you will encounter any pushback from any of your coworkers when this is implemented?
    - How would you overcome it?
- How do you know if you are doing a good job?
- What tools would you need to implement HPV vaccines here?
- What is the process like when making the decision to offer a service like this?
    - Are individuals identified to lead the effort?
        - If so, how are they chosen?

Collaboration Model Questions

- What do you think about the collaboration models?
- Which model do you think would be most successful? Why?
- What barriers do you see to implementing one of these models?

General Closing Questions

- If you were offered the chance to implement the collaboration now that you've heard all this, would you be interested? Why or why not?
- What would it take for you to be interested?
- What pharmacies in the area would you want to collaborate with? Why?

PARENT INTERVIEW GUIDE

Opening Questions for All Participants:

- How old is your child(ren)?
- Did your child(ren) receive the flu shot last year?
  - If yes, do you remember where they received it? Physician/pediatrician office, pharmacy, health department, school flu shot day?
- Has your child received the HPV vaccine?

For parents with child(ren) who have received HPV vaccine:

- How did you hear about the HPV vaccine?
- What kinds of things, positive or negative, have you heard about the HPV vaccination?
- Why did you decide to immunize your child(ren)?
  - Did anyone recommend the vaccine to your child(ren)? Who?
  - Did you consult with anyone (friends/family members) before immunizing your child(ren)?
  - Were there people out there who encouraged you or discouraged you from having your kids receive the HPV vaccination?
- Did insurance cover the HPV vaccination?
- Where did your child(ren) get the HPV vaccination?
  - Is that where they receive most/all of their vaccinations?
  - Who took the child(ren) to get the HPV vaccine?
  - Did you have to take off work to take them? For both doses?
- A child that receives the first does before they turn 15 needs 2 doses to complete the series while a child who receives their first dose at 15 or older needs 3 doses. When did your child(ren) receive their first dose?
  - Have they completed the series?
- How would you feel about having your child receive the series of vaccines at a local pharmacy?

For parents with child(ren) who have not received HPV vaccine:

- Have you heard of the vaccine? How do you hear about the vaccine?
- What kinds of things, positive or negative, have you heard about the HPV vaccination?
- You indicated that your child(ren) were not immunized against HPV. May I ask why?
  - Has anyone recommended the vaccine to you for your child(ren)?
  - Did you consult with anyone (friends/family members) when you decided not to vaccinate your child(ren)?
  - Are there people who are encouraging/discouraging you from having your kids receive the HPV vaccine?
- What kinds of questions would you like answered about the HPV vaccination?
- What are things that might convince you to decide to vaccinate your child(ren)?
- Do you know if insurance covers the HPV vaccination?
- Where would you take your child(ren) to get the HPV vaccination if you decide to get them vaccinated?
  - Is that where they receive most/all of their vaccinations?
  - Who would take your child(ren) to get the HPV vaccine?
  - Would you have to take off work to take them? For both doses?
- A child that receives the first does before they turn 15 needs 2 doses to complete the series while a child who receives their first dose at 15 or older needs 3 doses. How would you feel about having your child receive the series of vaccines at a local pharmacy?

For all parents:

- How would you like information about HPV vaccine to be delivered? Mail? Email? Text message? Television commercial? Billboard? Only in-person?
- What do you think would be the best way to remind a parent that it is time for their child(ren)'s vaccines? For example, since HPV vaccine requires at least 2 doses, how would you like to be reminded that it is time for your child(ren)'s 2nd or 3rd dose?
- What is your relationship like with your pharmacist?
- What do you think about having your pharmacist provide HPV vaccinations?
  - What do you think about having your pharmacist provide all vaccines for children and adults?
- Would you like it if your pharmacist contacted you to let you know that your child(ren) was due for a vaccine? Why or why not?
- Would you like it if your pharmacist talked to you about the vaccine while you were in the pharmacy? Why or why not?
- What would you think if your child(ren)'s physician referred you to the pharmacy for vaccinations?
